# Navigating uncertainty: exploring parents' knowledge of concussion management and neuropsychological baseline testing

**DOI:** 10.3389/fspor.2024.1360329

**Published:** 2024-05-10

**Authors:** Matthew Hagopian, Michael P. Jorgensen, Hugo Lehmann, Fergal O’Hagan

**Affiliations:** ^1^ Department of Psychology, Trent University, Peterborough, ON, Canada; ^2^ Faculty of Kinesiology and Physical Education, University of Toronto, Toronto, ON, Canada

**Keywords:** concussion, parents, risk, management, uncertainty, neuropsychological baseline testing

## Abstract

**Introduction:**

Parents play an important role in preventing and managing sport-related concussions among youth sport participants. Research indicates that parents understand the severity and consequences associated with the injury but gaps exist in their knowledge of its management. Neuropsychological baseline testing (NBT) is a modality that has gained interest in youth sport to purportedly better manage concussion injuries. Little is known about parents' perspectives on the use of NBT in the management process.

**Methods:**

The present qualitative study used Protection Motivation Theory as a guiding framework and employed focus groups (*N* = 2) with parents (*N* = 11) to gain insight into parents' perceptions and experiences with concussion management, specifically focusing on NBT.

**Results:**

Inductive Content Analysis developed a core theme of navigating uncertainty. Participants expressed uncertainty about the nature of concussion and its management process, where concussion was not always easy to identify, youth were not always reliable reporters, and there was no prescribed or proscribed path for recovery. Personal experience and concussion management policy provided participants with a degree of certainty in managing concussions. Participants gave NBT mixed reviews in potentially promoting greater certainty but also held reservations about its usefulness in concussion management.

**Discussion:**

We discuss findings relative to existing knowledge and theory in youth sport concussion and identify implications for practice.

## 1 Introduction

Sport-related concussion (SRC) is a complex injury (1), prevalent among youth and adolescent athletes. These injuries draw substantial attention due to potential long-term consequences (2, 3). SRC injury is defined as “a traumatic brain injury caused by a direct blow to the head, neck or body resulting in an impulsive force being transmitted to the brain that occurs in sports and exercise-related activities” (4, 5). The injury is experienced as a range of cognitive, emotional, and somatic symptoms, which typically resolve within 7–10 days. However, a commonly cited estimate from older literature (i.e., pre 2005) is that 10% of athletes in youth and adolescent populations that experience SRC will have an extended recovery period (1). Some recent research has shown that 31% of children and adolescents with acute head injuries continue to experience symptoms such as difficulty concentrating and headache beyond one month, affecting daily functioning and quality of life (6). These differences may be due to improvements in diagnostic systems, such as that developed by Zemek et al. (6). Symptoms present in response to neurometabolic rather than structural disturbances (7–9), making concussion difficult to identify, diagnose, and manage (1, 10, 11).

In Canada in 2017–2018, in excess of 100 SRCs occurred per 100,000 population in the 12–19-year-old age category (12). High-profile cases, like Rowan Stringer's catastrophic and lethal concussion injury (13, 14), have prompted legislative actions, such as Rowan's Law [mandating removal-from-play and return-to-play (RTP) processes for SRC in organized sports; (15, 16)], policy developments, like the Blue Card in rugby [a removal from play and RTP process following SRC in rugby; (17, 18)], and rule changes to enhance SRC risk reduction and injury management (19).

Neuropsychological baseline testing (NBT) is a widely promoted tool in concussion management, used in elite and youth sports, in US high school systems (20), and sport associations in Ontario, Canada. Although not mandatory, NBT provides a pre-season cognitive baseline for athletes, aiding in post-concussion management (4, 5, 21). This individualized cognitive profile allows clinicians to assess post-injury cognitive impairment, informing a tailored management plan. This can ensure that athletes recover before re-engaging in sports activities, reducing the likelihood of severe consequences such as second-impact syndrome (SIS) (22, 23). While NBT is theorized to enhance return-to-play decisions and mitigate second-impact syndrome risks, it is emphasized that it should complement, not replace, clinical findings in concussion assessment (4, 5). Although marketed as concussion management support, NBT is not endorsed for use (4, 5) and has limitations that warrant critique. In youth athletes, NBT may be of limited value owing to neurocognitive development and poor discriminative ability beyond the initial (i.e., post-24-h) period following a concussion (4, 5). Moreover, NBT demonstrates low test re-test reliability (24–27); this is particularly problematic in youth sports where cognitive development is dynamic (4, 5). Natural variability in cognitive abilities due to personal and contextual factors (e.g., academic stress) and purposefully underperforming (i.e., “sandbagging”) on baseline testing also compromises the validity of baseline testing (28–30). Overemphasis on baseline testing may overshadow other critical aspects of concussion management, such as graded RTP and learning and psychological readiness to return-to-sport.

Parents play a crucial role in managing youth SRCs, serving as advocates for their child's well-being and key decision-makers in the recovery process. Parents assume a *Case Manager* role by ensuring that their child adheres to recovery guidelines—taking sufficient time to recover before resuming sporting and academic activities (31). As case managers, parents must liaise with other sport participants on behalf of their child (i.e., parents, coaches, school officials) to ensure that precautions and accommodations supporting recovery are followed, to monitor their child's health status, and to make decisions based on their recovery progress. In this respect, the parent-coach-athlete triad [sporting triad, (32)] may have particular importance in concussion management in that each actor relies on the other for information on safe RTP.

In the event of SRC parents need to have access to knowledge of SRC signs and symptoms and the potential consequences of untreated injuries and be instructed to identify and implement effective management strategies to make recovery decisions that align with current guidelines. Research has shown that parents understand the severity of concussion but lack knowledge surrounding its identification and management (33–37). A lack of parental knowledge of concussion management strategies can lead to uncertainty in decision-making and potential problems for their child's health and well-being (e.g., delayed medical attention, adopting dated or ineffective strategies, rushing return-to-competition). This can increase the risk of further injury and the athlete's exposure to cumulative brain trauma [e.g., SIS; (14)] and long-term consequences [e.g., Chronic Traumatic Encephalopathy, CTE; (38)]. These potential risks, consequences, and implications for youth safety and well-being related to a general lack of parental awareness on SRC injuries highlight a need to examine parents' responses to youth concussions.

Parents, driven to minimize risks and support their child in sports, might be misled by technologies like NBT into a false sense of security. This may lead to deferred medical attention, assuming the technology suffices for professional medical advice or can somehow speed recovery. Allied health professionals marketing such tools appeal to parents' imperative to mitigate risks, creating uncertainty for those who opt out. In navigating this complexity, parents may unknowingly rely on unproven methods, potentially compromising their child's recovery. Understanding parental perspectives on such technology is crucial in mitigating these risks. We are unaware of research examining parent perceptions of NBT.

Protection Motivation Theory [PMT; (39)] provides a potential framework for understanding parents’ ability and motivation to respond to youth SRCs and engage with technologies such as NBT. In PMT, fear of health threats motivates behavioral responses through threat and coping appraisals (39). Threat appraisal considers vulnerability (i.e., perceived likelihood of a health risk) and severity (i.e., belief in the harm of short and long-term consequences, e.g., pain, impairment of learning) to enhance protection motivation. For concussions, this relates to parents’ beliefs about the likelihood and severity of their child experiencing a concussion. Also considered in threat appraisal are reinforcers that reduce the sense of threat, prompting maladaptive responses such as denial or minimization (e.g., belief that playing through injury builds character).

Coping appraisal involves self-efficacy (belief ability to enact protective behaviors) and response efficacy (belief in the effectiveness of the measure). Self-efficacy is influenced by experience, encouragement, and affective states (40). Parents' ability to identify symptoms and negotiate restricted play is relevant here. Response efficacy is influenced by experience, modeling, and education, such as concussion awareness programs. Response costs (weighing the costs of protective behaviors) are also considered. For athletes, removal from play is a direct cost. The money, time, and effort to administer concussion management are examples of response costs, as might be removing a child from play.

PMT is a useful framework to examine parents' responses to youth SRCs. PMT has been used in primary, secondary, and tertiary prevention to predict engagement in health prevention (41) and cancer screening behaviours (42), as well as rehabilitation and treatment participation (43, 44), and parents protecting their child's health (39). While not encompassing all potential influences, PMT provides a core of motivational and behavioural factors that might influence protective response, regardless of whether a child has experienced a concussion or not.

In sum, while parents possess sound knowledge of concussion injury, their understanding of concussion management is less certain, and limited research has focused on their experiences in this regard. This gap is particularly crucial given the rise of new technologies like NBT, positioned as solutions to the complexities of concussion management. Given the contested nature of NBT, careful consideration is needed regarding its inclusion (if at all), especially considering the influence of commercial marketing on parents who have a heavy emotional investment in their child's sport participation but may not have the expertise to evaluate such technologies (45). Our qualitative exploration aimed to shed light on parents' experiences, including their knowledge and beliefs about, but also their uncertainties and worries with concussion management, with specific reference to NBT, using PMT as a guiding framework. Our findings might help to underscore the uncertainty parents face, especially when navigating technologies like NBT, offering insights for future research and implications for concussion management.

## 2 Methods

### 2.1 Research design

A qualitative design was chosen due to the exploratory nature of this study and to present a rich and contextualized understanding of parents' experiences, beliefs, and feelings toward concussion management and their perceptions of NBT. Quantitative research may help identify parents' gaps in knowledge but offers little insight as to why these gaps exist or how they affect parents' experiences and decision-making processes concerning SRC management—elements that can be richly explored through qualitative methods. Ethics approval for the study was received from Trent University's Research Ethics Board in June 2020 (protocol #26630).

### 2.2 Theoretical orientation

Critical realism was used as the metatheoretical framework to identify generative mechanisms affecting human experience and action (46, 47). Critical realism recognizes internal experiences (i.e., thoughts, feelings) and social structures as influences while at the same time recognizing that those participating in the experience (including the researcher) play a role in constructing meaning and experience. This study examined parent beliefs, attitudes, and other features (e.g., legislation) as potential generative mechanisms for parents' concussion management behaviours. Critical realism does not deny the constructed nature of experience, but rather asserts that there is an underlying reality that we can only understand imperfectly. Critical realism also recognizes the importance of extending understanding into practical action, but emphasizes that effective action is best achieved by understanding the mechanisms of effect of a phenomenon. PMT (39) provided a sensitizing methodological framework to guide data gathering owing to its behavioural focus on risk management and development based on appeals to fear.

### 2.3 Context

The study was conducted in Ontario, Canada. Ontario enacted Rowan's Law in 2016, which formalized the obligations of sport communities to prevent, monitor, and manage concussions properly (15) as well as educate members of the community. Briefly, the legislation mandates that sport organizations educate community members about SRC and its management, monitor concussion incidents, and ensure that RTP and learning guidelines are followed in community sport and school settings.

Ontario has a publicly funded healthcare system where injured athletes can access emergent and primary care medical diagnostic and treatment services. At the same time, networks of private (i.e., for-profit) healthcare providers have grown up around concussion treatment. These networks market baseline assessment technology to clinics to allow the clinics to adopt the technology in providing their own concussion services to sport organizations. Ontario provides an ideal context to study parents, sport concussion management, and NBT, as sport and health care communities are taking active steps to improve concussion management, parents are highly involved in their child's sport(s), and NBT is widely distributed across the province.

### 2.4 Sampling and recruitment

Purposive sampling was used to recruit participants through advertisements posted on social media websites (e.g., Facebook and Instagram) and email communication with administrators of local sporting organizations (e.g., minor hockey and rugby). To ensure the relevance of concussion injury in soliciting parent experience, recruitment efforts targeted parents with children involved with contact and collision sports. Parents were excluded from the study if they did not have a child registered in contact or collision sport. Participants received a twenty-dollar electronic gift card for their involvement in the study funded through the fourth author's personal research funds.

### 2.5 Data gathering

Focus groups (conducted in December 2020) were chosen to facilitate dynamic discussions, allowing participants to compare their experiences (48, 49). Prior to commencement, participants submitted consent and demographics via the Qualtrics online survey platform. Participants were assigned to two groups based on online polling for availability. Groups were held remotely over Zoom, enabling participation during COVID 19 pandemic and providing a degree of anonymity and potential for greater openness about personal matters on the part of participants (50). Groups were recorded for transcription and analysis. The first author moderated the groups, the fourth author took notes and researchers debriefed after each session. The first group ran for 100 min, the second for 108 min.

The guide (Supplementary Material 1) used to facilitate focus groups was shaped by the constructs of PMT (51). To gain a sense of personal orientation, participants were asked what interested them in participating in the focus group before shifting to the first topic of questioning, which was their current beliefs surrounding concussion injury. Within this section, questions looked to examine parents' beliefs about SRC and the sources of those beliefs (e.g., what past education have you received regarding SRC?), their perceptions on the severity of the injury (e.g., in your mind, what are the consequences of SRC?), and what measures they viewed as an effective means of reducing the chances of SRC in children (response efficacy; e.g., rule changes, equipment).

The inquiry then transitioned to participants' experiences with the SRC management process. This phase sought insights into the actions participants took to aid recovery and their confidence (self-efficacy) in managing their child's SRC. Inquiries extended to interactions with medical professionals and recommendations for parents undergoing the recovery process for the first time. Further questions explored participants' experiences and opinions on NBT, probing its potential applications in SRC management.

As not all parents may have had exposure to baseline testing, brief media-based summaries of NBT were provided prior to the focus groups to orient the parents to NBT (Supplementary Material 2). Three media summaries were provided—one emphasizing the potential benefits of NBT, one its shortcomings, and one neutral focusing on the technical aspects of NBT. Media summaries were researched and derived from sports media and vetted by two independent researchers (one neuropsychologist and one philosopher) to verify the technical accuracy and orientation of the argumentation of the media summary.

### 2.6 Data analysis

Video recordings were transcribed verbatim by the first author and reviewed for accuracy by the fourth author. No transcription software was used. Inductive Content Analysis aligning with Hsieh and Shannon's (52) guidelines for conventional content analysis was employed for data analysis. Inductive Content Analysis was chosen for its suitability in exploring social phenomena with limited existing theory and prioritizing participant voice and lived experience over existing theory. Given the scarce knowledge about parents' perceptions toward NBT, Inductive Content Analysis was deemed appropriate. While PMT guided initial focus group questions, the analysis aimed to capture constructs beyond the theory, rather than seeking its validation as an explanatory framework for parents' management of SRC. PMT was used as a lens to discuss inductively developed themes.

Data analysis began with multiple transcript readings by the first author for data immersion. Initial impressions and comments were recorded, and key thoughts were highlighted through code labels (52). Themes were derived from multiple codes, and after developing themes and sub-themes for each focus group, comparisons identified similarities and differences across transcripts. The fourth author reviewed and discussed the evolving codes and themes with the first author, leading to the final construction of a common theme (“navigating uncertainty”). This theme encompassed sub-themes related to participants' experiences with concussion management and NBT. Examples were used from the text to contextualize and support the identified themes.

Our potential bias as sport participants in interpreting the data is acknowledged. To enhance rigor, independent researchers assessed the focus group guide for relevance and neutrality (e.g., ensuring questions would not lead participants to desired responding). The fourth author verified transcript accuracy (53). Multiple readings ensured analytical depth, and rich participant descriptions supported themes in the data. Throughout, we reflected on preconceptions to mitigate potential biases. The first and fourth authors actively contributed to theme development, fostering reflexivity (54), while the second and third authors critically reviewed themes to prompt further reflexive analysis (e.g., encouraging consideration of alternative interpretations).

## 3 Results and discussion

The results and discussion will first present the participant characteristics to help contextualize their accounts. We will then relate and discuss the themes in relation to extant literature and theory.

### 3.1 Participants

Table 1 provides a descriptive summary of the participants. As per the inclusion criteria, all parents had children involved in some form of collision or contact sport. The sample (*N* = 11) was gender balanced with a wide age range (23–61 years). Five participants were involved in coaching youth sport. Children were enrolled in a range of sports (i.e., hockey, rugby, football) from recreational to national level competition. Experience with concussion and NBT varied in the sample. Six parents had children who had sustained a SRC; four parents had their children undergo NBT. Three parents did not have direct experience with SRC or NBT. Participants were not known to each other.

**Table 1:** Participant demographic information.

Sports enrolled in and number of children per participants																			
Participants^a^	Gender	Age	Role of coach	# of children in sport	Hockey	Soccer	Baseball	Volleyball	Speedskating	Rowing	Ball hockey	Synchro swimming	Gymnastics	Rugby	Basketball	Track and field	Lacrosse	Football	Total Sports enrolled In
A1	F	47		3	X		X												2
A2*	F	52		2	X														3
A3*	F	48		2	X			X	X										3
A4*	F	43		2		X		X	X										1
B1	M	44	X	2	X									X			X	X	4
B2***	F	47	X	3				X						X	X	X			5
B3	M	23		1						X				X					1
B4*	M	61	X	3		X			X	X									3
B5*	M	45		3	X	X	X												5
B6***	M	53	X	2	X								X						1
B7**	M	39	X	2	X						X	X							3
		Total	5	25	7	3	2	3	3	2	2	1	1	3	1	1	1	1	

^a^ Participants were given codes in replacement of their name to maintain confidentiality. The letter represents the focus group they were a part of, and the number is respective of their position in said focus group.

Participants with a * have had a child experience a sport-related concussion, ** has not had a child experience a sport-related concussion but has engaged with neuropsychological baseline testing, *** has had a child experience a sport-related concussion and neuropsychological baseline testing.

### 3.2 Thematic summary

Our analysis resulted in five sub-themes that clustered around a core theme—navigating uncertainty. Navigating uncertainty encompassed our participants' efforts to manage their child's concussion in the face of an incomplete understanding of the injury and its management process. Participants had to learn and adapt while being presented with new and challenging experiences.

**Figure 1:**
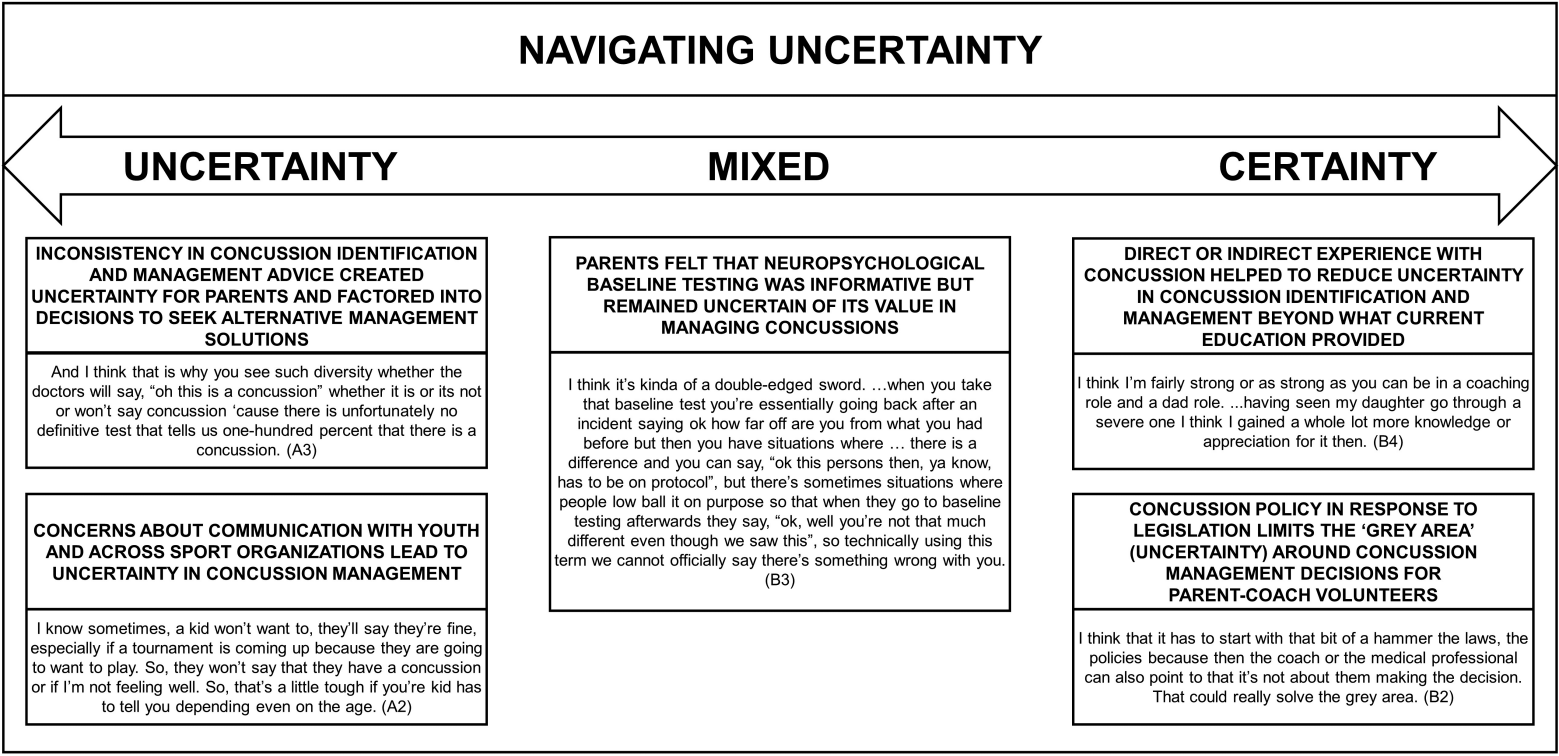
Summary of themes.

Among the five sub-themes, two reflected an orientation towards greater uncertainty, two reflected an orientation towards greater clarity, and one reflected ambivalence. Uncertainty prevailed regarding the elusive nature of concussions, challenges in identification, and the absence of a standardized recovery path. Personal experience and concussion management policies offered a measure of certainty. The evaluation of NBT as a tool for enhancing certainty in concussion management yielded mixed reviews. We represent this bidirectional influence in Figure 1, providing an illustrative quote to support each sub-theme. The core theme and each of the sub-themes are elaborated below, with reference to extant literature, and integrated with theory.

#### 3.2.1 Navigating uncertainty as a core theme

Participants were cognizant of and worried about the severity of concussion injury, identifying potential short- and long-term consequences and the vulnerability of their child to those effects.

“… people are much more aware of concussions and talk about it more. So, ya I think parents are very worried about the long-term consequences because, you know, the fear is the more you have the more damage you could be doing to your child or child could be doing to themselves. So, ya I think concussions are a big concern.” (A4)

Perceptions of severity and vulnerability were accompanied by participant recognition of their central role in managing both concussion prevention and management, while also implicitly acknowledging the value of sport for their child's development.

“As parents, now we know more about concussions then we've ever known in the past and it's ultimately our decision to sign our kids up for whatever sport it is that they want to participate in and unfortunately a lot of it falls on the parents right now, you know, to make sure that we're guiding them in the right direction and we are looking out for their best interest…” (B7)

In other words, threat perceptions prompted these parents to engage with concussion management vs. minimizing or denying the potentially harmful effects of concussion to reduce anxiety. At the same time, parents were reluctant to have their child miss out on sport and the development opportunities afforded by their involvement in contact and collision sport.

Examining navigating uncertainty as a core theme within parents' decision-making through a PMT lens, threat appraisal reflected a balance between risk and reward. The threat component of concussion was present in acknowledging the potential severity of concussion injury; however, parents were willing to risk exposure to sport situations that may leave their child vulnerable to concussion owing to the benefits of participation. Parents identify significant cognitive (e.g., need to maintain grades to participate) and social (e.g., social cohesion and responsibility) benefits from participation in high-risk sport (55). This points to a more nuanced view of injury threat as it applies to sporting contexts. With PMT's prioritization of health outcomes, one might construe parent's willingness to allow their child's exposure as “maladaptive” in denying their vulnerability. However, it is clear that participants weighed the risks and benefits and were attempting to mitigate and consider SRC risks when allowing their child to participate in sport. In contrast to these considerations made by participants, another work indicated some parents believe there is too much attention placed on concussion injury (56), which reflects a minimization of SRC severity.

#### 3.2.2 Inconsistency in concussion identification and management advice creates uncertainty for parents and factors into decisions to seek alternative management solutions

Participants entering the concussion management process were required to have their child medically assessed. They expected this assessment would lead to a definitive medical diagnosis to provide direction on next steps in management.

“…so what's the truth here like how do we decide what is a concussion if the medical field, like, I'm not a doctor, I can't diagnose my child and I'm totally relying on the medical field to tell me if my kid has a concussion.” (A2)

This expectation was not always realized. Participants perceived inconsistency and ambiguity in concussion identification, mainly during interactions with emergency room physicians. Also contrary to what might be expected, these differences in experiences were present within the same hospital emergency department. For A3, they had experienced no diagnosis and received minimal supportive materials for both her child's and husband's concussion, while A2 had a doctor who provided them with the diagnosis and supportive information (e.g., brochure) they needed to aid in their management process.

“So, the fact that A2's daughter was asked to come back to the doctor after three days, I mean that was great. We didn't unfortunately have that guidance. I again think it's that lack of consistency, right. Ya know, some doctors are really focused and wanting to get, ya know, whether it's a definitive diagnosis or give them the information they need to get better whereas others are kinda like ‘well it might be, so just so you can see what happens’, kind of thing.” (A3)

“Ya I found they were really great and very caring, but I also find that not everybody is delivering the same message to everybody who comes through the door, so that's a little confusing. If you go to a different physician or different emergency room or clinic, you might get a different message.” (A2)

The lack of diagnosis has been previously reported by Boutis et al. (57) who found that emergency room doctors were diagnosing concussion in children less often relative to the Zurich International Consensus Guidelines (58) suggesting a more hesitant diagnostic stance. Of the 495 cases that they examined, only 200 were diagnosed as concussion by the emergency room doctors while 443 of the cases were judged to meet the criteria for the Zurich guidelines. Diagnosing a SRC is not an easy process considering there is no definitive test and symptoms could take days to develop (11, 59). This leaves emergency room doctors to rely on signs and symptoms that they see during an initial assessment alongside information from the athlete and their parents, which may not, at that moment, lead to a diagnosis of an SRC.

Knowing the difficulties associated with diagnosing a concussion, it is important to understand sport participant expectations in an urgent care consultation for a suspected SRC. Zamarripa et al. (11), surveyed parents' expectations and beliefs surrounding concussion diagnosis in an emergency room setting, concluding that parents were expecting more than what the emergency room doctors could provide. Parents expected comprehensive and definitive care, including imaging, a definitive diagnosis, a timeline for return-to-activity, and a signed RTP form (11). In practice, emergency room doctors review the patient's SRC history and previous conditions (e.g., learning disorders, migraines, mood disorders), rule out any severe injuries that may need imaging (e.g., cervical spine injury), and potentially administer an age-appropriate symptom inventory [e.g., Sport Concussion Assessment Tool—5th Edition, SCAT5; (60)]. While participants might perceive the absence of a concussion diagnosis as a shortcoming on the part of healthcare providers, it is imperative to acknowledge the inherent limitations of the initial assessment, particularly given the constrained timeframe and the necessity for symptoms to manifest.

For the participants who did not receive a definitive diagnosis, they were left without an understanding of what exactly their child had experienced. This uncertainty created a sense of doubt for how they were going to approach their child's injury.

“I think when my son had his first concussion, it was a bit of a learning curve of ‘does he have it, does he not have it?’ and not fully understanding…I think going through it the first time it was kind of easing in [to managing the concussion].” (B5)

Perceiving a lack of clear direction and in the absence of experience, participants managed concussion according to what they felt would benefit their child's recovery, mainly relying on their child to express their symptoms to understand how they were feeling and supervising and limiting their child(ren)'s daily activities accordingly.

“Lots of sleep, trying to minimize media, which in this day and time is really challenging because, I mean, even school, like they're using smart boards and they're using digital everything. We let him lead the way in terms of, we wanted him awake during the day just a little bit, we [just tried to help him move a little bit] say lets go for a walk or something and we just listened to his cues, if his head [was starting to hurt] he would go back to bed.” (A3)

The uncertainty in management led three of the participants to access auxiliary health care, including unresearched therapies or trainings, influenced by existing relationships they had with healthcare providers (vs. evidence-supported interventions recommended by a healthcare provider). Two of these participants felt their child benefitted from the added care, although the methods (i.e., craniosacral therapy, virtual focus training) they pursued were exploratory and not evidence-based.

“We have a friend who's a neuroscientist who works at the university and he was conducting studies with people who have suffered concussions, so we were able to get my kid into that trial which was great. But he did a lot of work with focusing training and it's almost like a video game, ya know, and that really helped him recover quite quickly.” (B2)

A3 described taking her son to a colleague who did craniosacral therapy, which seemed to help resolve his residual symptoms. Whatever the efficacy of the interventions, for parents these alternative methods seemed to be attempts to exert some form of control over a management process they did not fully understand.

Thus, parents were more confident in their immediate actions (e.g., reducing screen time), but less certain when it came to managing prolonged symptoms. Perceptions of diagnostic ambiguity coupled with unrealistic expectations of a clear pathway from identification through management undermined participants' efficacy in managing SRC.

From a theoretical standpoint, this sub-theme reflects how the difficulty in pinpointing the nature of threat can create confusion in response. While PMT does not address this aspect of threat, the Common-Sense Model of Illness Representations (61) elaborates how different dimensions of injury or illness representations might affect one's coping response. In this framework, having clarity in the identity (i.e., associated signs and symptoms) is important to one's response. Where the symptom picture is ambiguous and individuals are uncertain about personal control a problem-focused coping response is less likely (62).

Where uncertainty did play a role was in the response efficacy of longer-term management strategies. Lacking guidance, some participants turned towards unproven and experimental interventions to support management and were willing to consider NBT as it was a “better than nothing” approach. Through a PMT lens, this response is motivated more by the benefit of its anxiety reducing effect than on any beliefs held about the response efficacy of the modalities in question. Anxiety reduction can be looked at as a response benefit, while maintaining anxiety in the absence of action can be looked at as a (non)response cost.

While this might seem innocuous, as there is likely no harm in exposure to modalities such as craniosacral therapy, (a) it directs resources, energy, and attention away from potentially beneficial management methods like progressive aerobic exercise, and (b) it serves mainly as a form of emotional coping rather than addressing the actual danger posed. As SRC most commonly resolves over time, the coincidental resolution of symptoms with the application of such techniques may lead to the anecdotal belief that the intervention was effective when research does not currently support such an approach.

#### 3.2.3 Concerns about communication with youth and across sport organizations leads to uncertainty in concussion management

Participants relied on communicating with the athlete alongside other actors (e.g., teammates, coaches, administrators) within the sporting community to manage a suspected concussion. The need to trust a young athlete about their condition worried parents because of the internal and external factors they believed could influence their child into hiding or inaccurately reporting their symptoms—concerns aligned with extant research in youth athletics (63, 64).

Parents acknowledged their child as a committed sport participant and knew that they would not like to miss games or practices. This can make it difficult when trying to identify or manage a concussion. A2, for example, expressed concerns about their child not disclosing a concussion to not miss out on play.

“And I know sometimes, a kid won't want to, they'll say they're fine especially if a tournament is coming up because they are going to want to play, so they won't say that they have a concussion or if I'm not feeling well. So, that's a little tough if your kid has to tell you, depending even on the age. So, that's, we found a bit tricky, like our daughter would go full-tilt all the time and I'm not sure that some kids wanna miss.” (A2)

Echoing prior research, young athletes fear repercussions, including potential playtime loss, if honest about their symptoms (29, 65). To avoid perceived punishment, athletes may withhold information, indicating a possible overconformity to the Sport Ethic (66). The Sport Ethic encompasses normative beliefs like making sacrifices, accepting risks, and playing through pain. Overconformity manifests as athletes uncritically embracing and committing to these norms, potentially leading to deviant behavior, such as withholding health information (64).

Alternatively, young athletes could also be limited in providing information because of difficulties identifying and communicating what they have experienced as a potential SRC (67). A3 reported that when her child experienced a concussion, they did not have the ability to properly express their symptoms.

“Kids don't always have the right words either, right? They don't have those like necessary skills, sometimes giving them like I gave him wording like ‘there's pressure in your head, is there something sitting on your head, do you have a headache or do your eyes hurt?’ Like I gave him those things to kinda go through just again cause I know a lot of the weird symptoms that can happen with concussion.” (A3)

Unlike other sport-related injuries, SRC has a range of complex signs and symptoms that can occur hours up to days following the initial incident. The delayed onset and other known explanations (e.g., dehydration) could lead to athletes questioning if what they experienced was a SRC and if it was serious enough to report (63). Past research on collegiate athletes in the U.S. performed by Kaut et al. (65) found that nearly 32% of their sample reported experiencing a blow to the head that led to subsequent symptoms of SRC but continued to play due to the inability to self-identify their symptoms as a concussion. Similar findings were documented by Cusimano et al. (63) who interviewed 31 minor hockey athletes and found that underreporting of SRC was partially caused by the inability to recognize their own symptoms. There may also be denial on the part of the concussed athlete, believing what they want to be true (i.e., they don't have a concussion) to avoid the negative consequences of sitting out. In sum, parents were uncertain that their child could or would provide reliable information to enable them to properly manage a concussion injury.

Developmental dynamics are also a potential consideration for parent-child interactions. Research suggests parental monitoring can protect at-risk youth from peer pressure to engage in health risk behaviors (69). However, as children mature into adolescence, they may become more recalcitrant in discussing personal issues with parents (70). This was a concern for parents in another study (71) and is consistent with developmental literature on parent-child dynamics (72, 73).

Social influences may also play a role in concussion reporting. A4 experienced this firsthand with peers from her son's hockey team pressuring an injured player to try and play during an important game, also revealing the important role of parents in managing injury.

“…some kids might not be truthful leading up to a tournament or something where they really want to play a certain game and I have experienced that firsthand with one of our teams. We had a peewee team who their key player was injured, and the entire team wanted that player to be there, but that player very obviously was injured, and it was the parents who actually stepped in and was like, ‘nope you can't play’, and it was a very important game for that team.” (A4)

External pressure is a common feature of competitive sport caused by a sporting culture that reacts negatively to injury disclosure (63). Kroshus et al. (74) reported on a survey of 328 U.S. collegiate athletes, 26.5% of whom experienced pressures to remain in play from teammates, coaches, and parents. Frey (75) and Nixon (76) identified that the motivation for athletes to play through injuries is a socially learned behaviour. They suggest it is reinforced by the accolades of “playing through pain” and avoiding social disapproval (“come on, suck it up!”), and it is taught at a relatively young age (77). As a hinderance on the effectiveness of sport legislation such as Rowan's Law, the negative culture surrounding symptom reporting continues to be a cause for concern (78).

The lack of information exchange between sporting bodies was another source of uncertainty for parent-coach participants. As is evident in this sample, children play multiple sports, at varying levels and formats of competition. Parent-coach participants were concerned that they may be playing athletes who did not disclose concussions they experienced while participating in other sports competitions or trials (e.g., summer league tournaments) and therefore not have their injury managed adequately.

“Like if I coach a kid in a spring team but he's from another town and he suffers a head injury I can send him home or off the ice all I want, but when he goes back to his regular team, there's no one there that might know that this has happened and hopefully his parents, ya know, are looking out for his best interest, but unfortunately, that's not the reality in every case.” (B7)

B5 shared a direct experience of this where a parent did not feel the need to report an injury (a broken arm in this case), which led to an injured athlete continuing to play and risking more severe injury. B5 stated, “we were told nothing about it… it's a tough situation for various sports coaching when you're not informed by the parents.” Parent-coach participants were worried of potentially playing an injured athlete because of a lack of knowledge surrounding the health status of the athlete in question.

Youth sport's main social system, the sporting triad (coach, parent, and athlete), operates with each member responsible for their role to ensure success (32, 79). Disruptions within these triads can detrimentally affect the youth athlete's experience. One participant encountered such disruption when a parent failed to disclose their child's injury from another sport, leading to the athlete playing despite the injury's severity, turning a minor issue into a significant one. Withholding information may reflect the adoption of a professional model for their child, potentially becoming overly emotionally invested in their child's sport (80). Excess emotional involvement can skew their perspective on balancing health and performance in the developing youth athlete, leading to risky behaviors like withholding information from the coach. Such actions can contribute to a normative culture within the team that undermines injury reporting (80, 81). Previous research supports these assertions as parental pressure to achieve (82) and excessive identification with sport (83) influence parental response to concussion communication.

#### 3.2.4 Direct or indirect experience with concussion helps to reduce uncertainty in concussion identification and management beyond what current education provides

Participants reported either directly managing or vicariously witnessing the management of a SRC as a valuable learning experience that they could not gain through education alone. Their experiences provided them with an insight into the complexities associated with the injury and its management process.

“The personal experience is really valuable as well, ya know, even if someone hasn't experienced it ‘cause we can all attest to the fact that the proximity that you have to this kind of experience the better understanding and appreciation you have for the severity of it [concussion].” (B2)

These complexities were described by the participants as factors that would not be well understood by those without experience managing the injury. The participants noted that the “invisible” nature of concussion would make it difficult for those without experience (direct or indirect) to understand and appreciate the severity of the injury as experienced by their child.

“I think the problem with concussions is that unlike a broken arm, there's no label that there's something wrong and people don't and parent's and fellow athletes don't necessarily appreciate all of those symptoms that have been talked about so far.” (B4)

By extension, the ability for concussion injuries to cause irregularities in an athlete's mood, behaviour, and psychological well-being needed to be experienced by being around the individual during the management process. Participants reported changes within members of their own social circle that affected their family life, profession, and schooling.

“Well the social emotional part of a concussion that people that haven't been around concussions really don't know that it's just not physical, it's just not the fact that you have blurred vision or difficulty seeing with light and have headaches and all the rest, it's the social emotional bit. Like, I had a friend that their son had a bad concussion and he literally became a different boy for six months. He became very moody.” (B1)

The grounded understanding gained from experiential learning extended participants' understanding beyond that provided by public health education by expanding the parent's view on the scope of the injury's effects and reducing the novelty of management. The value of experiential knowledge contrasts with the general passive educational strategy from healthcare providers, such as pamphlets detailing concussion signs and symptoms. A systematic review by Curran et al. (84) revealed that most information provided to parents in pediatric emergency care used passive dissemination strategies. Rice and Curtis (36) underscored the limitations of passive education, noting that parents exposed to such programs struggled more with identifying mood and sleep symptoms compared to cognitive and physical symptoms.

Participants also indicated that current education efforts in the sporting community lacked specificity and organization. Their first criticism was that information was directed mainly to coaches and trainers instead of the parenting community—parents were not being provided with the educative support they needed even though they were the ones that had to manage their child's concussion.

“I think awareness overall is the big thing that needs to come out more for most parents. I know there's been a lot of awareness, like lots of literature towards coaches and coaching staffs and trainers, but the general population of parents haven't been shown as much of this information and I think getting it out there and the awareness to the parents, so they can realize what concussions really are.” (B5)

Supporting this claim, a systematic review of concussion education programs found none tailored for parents of youth athletes (34, 35). Despite parents' pivotal role in managing SRC, resources primarily emphasized identification and awareness over management (34–36). In jurisdictions mandating concussion education, research showed that only 16% of parents received education from coaches, with the majority (58%) merely signing an information form. Similarly, in Ontario, 42% of schools provided concussion education to parents, while 52% had parents sign a participation form including concussion information (85). However, the education was generally passive and there is no indication of parents' engagement with the provided information.

Their second criticism was with the organization and consistency of information that is already accessible for parents. They did not feel confident in choosing or following any specific option of care due to the lack of centralization and consistency surrounding opinions on concussion and methods used to manage it.

“I wish there was just a one stop shop for ‘this works’ and ‘this is what we should be looking for’, but because like anything you just punch it into the internet and, you know, it's find the information that you're actually looking for, and I guess what I'm trying to say is I just wish it was more cut and dry. There was more, ‘hey let's go to concussion.com, let's go to concussion recovery.com’, whatever it is, instead of just Joe's concussion recovery… there's just so many people that have an opinion or information or have done research and sometimes it varies from person to person.” (B7)

The value of experience described by the participants demonstrates how experience increases self-efficacy for concussion management. PMT identifies self-efficacy as a key motivational element and mastery experience as the most effective way to build self-efficacy and reduce uncertainty (40). Theories of experiential learning (86) elaborate how experience contributes to self-efficacy development, where learners benefit from: (1) firsthand, concrete experience with the symptoms and management challenges, with (2) reflective observation over time to deepen understanding that (3) enables abstraction of conceptualization to synthesize a multifaceted view of the nature of recovery (e.g., personality and mood changes) and (4) allows for experimentation with supportive actions (86).

The information provided by public health initiatives is essential to sensitize parents to concussion threat and to provide information about the basic tenets of concussion management (e.g., acute rest) and how to access further support as needed. It may not be beneficial or practical, however, to provide parents with the depth of insight required for longer-term management, such as would be gained by direct experience. In this case connecting parents to the experiences of peers who have already been down the path, through question-and-answer forums or social media may help parents to tap into knowledge that is tailored to their child's specific needs.

The concussion prevention and management landscape is constantly evolving. There remains debate around the reasonableness of the demands placed on parents in terms of knowledge and skills for concussion management (87). A Delphi panel of health care experts indicated that one-third of parent behaviours that were deemed important, were not viewed as realistic. Further to this, the expert panel identified only 7 of 24 necessary knowledge domains for executing these behaviours to have adequate scientific consensus. If experts are uncertain about the scientific foundations for concussion management, how can we expect parents to make decisions in an environment where there is significant grey area? Despite the central role of parents in concussion identification and management, it seems that parents are in a peripheral position when it comes to concussion education and the logistics of meaningfully educating parents remains a challenge (85).

#### 3.2.5 Removing the “grey area”: concussion policy limits the uncertainty around concussion management decisions for parent-coaches

Parent-coach participants viewed developments in sport-specific policies (e.g., Hockey Canada's concussion policy) in response to legislation requirements (e.g., Rowan's Law) as a benefit because they reduced uncertainty within the concussion management process. Protocols reduced what these five participants deemed to be the “grey area”. These policies allowed for parental decisions to be supported by a formal guide to help identify potential concussion events, guide concussed athletes through the RTP process, manage their interactions with parents, and raise SRC awareness.

“Like Rowan's Law and a little bit of awareness in the community and in the sports community has made my life as a coach way easier. Soon as it's, ok there was clear contact to the head, one or more symptoms, you're done for the day… talk with the parents afterwards, here's the form, they need to see a doctor, it's out of my hands, we're all just trying to keep your kid safe. I think it's like the grey areas have been removed, which, I mean, as long as we all sort of live to the letter of Rowan's Law, a lot of the grey areas have been removed. So, I feel actually a lot more comfortable now than I did five, six years ago. Again, I coach football, rugby, and hockey and there, people do get bumps and bruises and knocks in the head and it's a reality.” (B1)

Athlete removal policies and RTP guidelines simplified the decision-making process for coaches by providing a standard, evidence-based framework for how to support athletes during their recovery. Concussion policies reduced pressure on parent-coaches by providing mandatory processes, minimizing reliance on individual judgment when questioned about athlete removal decisions by parents. “The coach or the medical professional can also point to that it's not about them making the decision.” (B2). Concussion policy gave the parent-coaches a sense of security knowing that parents and athletes had to abide by the policy to RTP.

“My wife actually coaches as well and last year she had a player that was, their trainer thought had a concussion. They had to go to the doctor, get the note signed, the doctor said that they had a concussion and then before they actually got the letter re-evaluated for the doctor to sign off, the parent was trying to force the coach to make the player play and my wife was like, ‘No. She, that player cannot play until you get the doctor's note signed’, and I think that's a good step.” (B5)

The pressures coaches experience from parents to return their child early may stem from a lack of knowledge of the mandatory RTP protocols on the part of parents or, more concerning, knowingly trying to return an athlete to play before they are cleared. Hecimovich et al. (88) found that, of the 1,441 parents sampled, a high percentage (95%) understood that athletes should be removed from play following a suspected concussion; however, less than half of those parents (41%) endorsed a gradual RTP guideline for recently concussed athletes who are symptom free.

Although not expressed in this sample, parental pressures encouraging their child to play through injury could be viewed as an attempt to circumvent concussion protocols, which these parents may believe undermine parental autonomy over their child's care (82). Consequently, this may position parents in opposition to the coach and the removal decision. Research describes how parents act on their negative perceptions of coaches through contrarian action [e.g., resisting a coach's decision, questioning the actions of the coach; (89)]. Black et al. (33) reported that of the 786 youth hockey parents sampled, 15%–20% reported that they did not consult a physician for assessment or clearance to RTP following concussion and 19% stated they would not actively seek care from a physician for concussion management guidance. Whether through ignorance or contrarian and competitive attitudes, policy helped support removal and RTP decision-making on the part of parent-coaches. Alternatively, this issue may not simply represent contrarian behaviour on the part of parents. Limited access to primary care in certain jurisdictions (e.g., Ontario, Canada) makes the requirement of a doctor's note an important systematic barrier that limits the equitable application of such policies (78).

While not an element in PMT, policy also has a norming property that can change the standard operating procedures within a community. From a theoretical perspective, policy can help to reduce the distortions that might occur in motivation towards proper concussion management by decreasing the intrinsic and extrinsic reinforcers/aversives that encourage maladaptive behaviour. The fact that a policy exists to remove and rest means that teammate and coach encouragement to play on or the child's fear of missing out is less likely to be acted on to subvert the RTP process. Policy also promotes response efficacy by pointing those involved towards efficacious action (e.g., graduated RTP protocols with monitoring) and away from responses that are ineffective. Clear guidelines and standards establish expectations within a community that those involved may be reluctant to disregard for fear of sanction or social disapproval. Eventually, these standards can become internalized as responsible and ethical behaviours on the part of sport community actors.

#### 3.2.6 Parents feel that neuropsychological baseline testing is informative but remain uncertain of its utility in managing concussions

NBT use generated conflicting opinions among participants when discussing its value within the management process. Some parents questioned the utility of test results, while others felt that its inclusion could provide clarity (certainty) for the management process. The need for certainty was connected to the belief, for some, that using NBT would be better than doing nothing at all.

“I think any extra tool that we can use at this point to try and diagnose a concussion is very helpful because like it's an invisible injury for the most part and I think that's the biggest troubling thing for most parents and the athletes themselves is any information is good information.” (B5)

NBT was seen as a tool to make concussion more “visible” and bring some objectivity to its identification. In the absence of a definitive diagnostic tool and despite issues of reliability, it was also thought that NBT serves to reduce troubling uncertainty.

Three positions were introduced by participants to justify decisions to use NBT. The first position was the decision to engage with measures they were not confident about but were willing to include if there was a chance of the test possibly helping.

“I could see people doing it because, ya know, why not? Is it helpful? Maybe, maybe not, but if it was included in as a team thing and the coach and trainer said, or the league said this is what we're doing this year, then I think people would be on board with it because, ya know, it's not a big deal, it's not a cost, not a hassle and maybe it will be helpful, but we don't know, right?” (A1)

This position was advanced by parents whose upside belief of NBT was limited, but saw little downside, irrespective of evidence for use.

The second position was the ability for NBT to generate tangible evidence for SRC. This related to the visual results the test provides to parents and administrators to reflect on during the identification and management process. Although research has shown the results of NBT's lack reliability and should not be used in a standalone fashion in a clinical decision for RTP (4, 5), its ability to provide tangible results was enough for three participants to feel comfortable including it in the management process.

“I think in theory it's a great idea that we have something measurable and tangible because concussions are so fuzzy for us, to have something measurable to say your score was ‘X’ beforehand and it's now x minus 10, you're not up to where you were cognitively beforehand. So, I think that to make an effort to have something measurable is beneficial.” (B1)

Thirdly, participants noted that, while providing speculative objectivity to the SRC management process, NBT could function as an educative tool for the sporting community.

“But I think it's important to have that information, the concrete information embedded somewhere, but it also highlights, it gets people talking, right? It also calls on people to take responsibility and be involved and understand what, ya know, it's like in coaching where we're expecting our coaches to have a criminal record check and they have to go through some sort of process, right, to be tracked, to be part of the community. … Like it functions as an education, it functions as an awareness.” (B2)

In this sense, NBT requires behavioural engagement by the parent, coach, and young athlete (84, 90) providing another element of exposure to SRC education which the participants felt parents, coaches, and athletes could benefit from. It is also a means to promote parent-child communication about concussion, something that has been recommended in previous studies (82). NBT may also provide opportunities to engage actively and collaboratively with a concussion-focused modality, vs. passive engagement through single administration methods (e.g., brochures). Thus, justification for NBT depended on how the participants viewed the function of the test itself (i.e., diagnostic vs. educative).

On the other hand, participants had significant reservations about the inclusion of NBT in the concussion management process. Consistent with concerns raised in research on NBT (4, 5), they understood that accurately recording and comparing their child's scores months apart would not be an effective way for identifying or managing a SRC. Having the existing gap in testing during the developmental years of a child's life would make the pre-season assessment void if the injury were to occur months later. They would then be left to deal with a SRC and no test to help with the management process.

“even if you have a baseline and I get that the idea is that if a kid has a concussion they're checking them to see if they're back at that baseline, like I said, kids change. So, say they had their baseline done and the concussion happens even eight months later, the baseline could have changed in between that time and they don't know.” (A1)

Participants struggled to justify incorporating NBT into their sport organizations, especially when they already had comprehensive RTP protocols in place. Additionally, the added expense and time commitment of NBT seemed unattractive, considering its perceived redundancy alongside existing, mandatory youth sport RTP policies.

“So, why not just treat the concussion and make sure it's gone and make sure they're better before letting them go back and making sure that they're passing all their new whatever milestones or getting better? They would have to do that regardless of if they had a baseline. So, it's kind of like, what's the point other than sounds like it's a chiropractor money grab.” (A1)

The lack of preventive benefits from NBT further fueled doubts: “it's not going to prevent anything [concussion]” (A1). Instead, participants favored a more direct approach, targeting treatment for the injury according to established guidelines, deeming it a more effective management method than investing in a costly auxiliary test.

Consistent with concerns raised in the literature (28–30), participants were also concerned with the ability for young athletes to purposefully score lower on a NBT to try and RTP faster. B3 expressed these concerns with NBT as a strategy because of the way young athletes could game the system knowing that the baseline scores they provide would make it easier for them to pass the test if they were to suffer a SRC.

“But there's sometimes situations where people low ball it on purpose so that when they go to baseline testing afterwards, they say, ok, well you're not that much different even though we saw this. So, technically using this term we cannot officially say there's something wrong with you.” (B3)

B4 supported B3's claims discussing how the assessment may become vulnerable to sandbagging (i.e., purposefully underperforming on the test) when providing the young athletes with information on how the test is supposed to fit into the SRC management process. They believed that once the athletes are provided with the information on how the process around the assessment is supposed to work, athletes would purposefully use that knowledge to underperform their test to guarantee a result that would allow them to RTP.

“They sandbag it. ‘Cause when you educate them, which you need to educate them, they figure out, well, I need to do poorly on this test to guarantee that myself I can get back to competition if I do get a concussion.” (B4)

The idea of young athletes purposefully scoring lower was not a matter of if but rather when they would try to cheat the test. B4 felt that athletes within his own sporting program would try to score lower based on his ongoing interactions with them in a sporting context “Well I know some speed skaters that would put a fix in on the test”. B2 supported B4's claim from experiences that she had witnessing young athletes complete NBT, “I've seen kids game it for sure”. Participants, including those who did not hold coaching roles, knew that some youth athletes would alter their scores to RTP faster. The chance that a player may be able to circumvent the assessment added further doubt and uncertainties for the inclusion of NBT in their league's current RTP protocols. Knowing that they already had a structure in place that required time away from play and a final sign off by a physician made them question the purpose of including an assessment that would not benefit the management process. Sandbagging is not a speculative concern. In a retrospective study among a sample of 6,346 high school athletes, Tsushima et al. (91) classified 47% as having sandbagged performance with underperformance affecting pre-post comparisons among concussed athletes in the sample.

Looked at from the perspective of response efficacy, it was encouraging that, when presented with information on NBT and arguments for and against its use, participants adopted a more critical view of its inclusion in concussion management, also acknowledging the value of current return-to-play procedures. This more rational perspective aligns with current recommendations about the inclusion of NBT in the management process in that it should only be used in conjunction with proper medical assessment and RTP procedures. Apart from the limitations of NBT in research findings, requiring its use can create equity and access issues in sport to families and communities who may not have the resources to afford baseline and follow-up testing (92, 93). For parents who are able and opt to make the financial investment and utilize NBT, their child may feel greater parental pressure as a result, which can impact their enjoyment and commitment to their sport (94).

## 4 Strengths and limitations

While our sample was small and non-representative (of both all sporting parents and parent-coaches), the information gained was complex and insightful. The focus group format and duration supported rich discussion and sharing of experiences among parents, emblematic of the peer-oriented and experiential approach we propose in the following section as a potential education intervention. Qualitative responses captured a range of experiences that have been shown in the literature including around diagnosis by a medical professional (11, 55) athlete underreporting and hiding symptoms (63, 64) and concussion management experience and education, or lack thereof (33, 36, 84). Our findings provide novel insight into parents' decisions on, and navigation of, the concussion rehabilitation process with their child(ren). These findings decentre the focus from parent knowledge towards the cognitive/affective experience of parents managing SRC without full information and in a complex environment where actors hold potentially competing priorities and views. Additionally, our study sets the stage for future research to examine specific areas highlighted by our themes (e.g., communication networks and flow of information within and across sport organizations and the impact of sport policy and experience on parents' care decisions for their child athletes).

We studied parents in Ontario, Canada, a province with concussion management legislation and policies within the administration of sport to guide actors involved in concussion management. While these findings may not transfer entirely to jurisdictions without such structures, parents' uncertainties of managing the concussion process will likely have resonance in other contexts. Future research could look to expand participant recruitment across Canada and other sporting contexts. We were also aware of the limitations of NBT as a tool for identifying SRC when initiating the research. We acknowledge the potential for researcher bias; however, we attempted to manage our pre-existing beliefs through a reflexive and systematic approach. We also assert that a critical approach to research inherently carries values towards empowering participants toward positive change in the field of study.

## 5 Conclusion and implications

Our study of parents' experiences, beliefs, and attitudes towards concussion management and NBT provided insights into influences that moved parents towards greater (personal experience, policy) and lesser (diagnostic ambiguity, communication) certainty around concussion management. Views on NBT were mixed in recognizing potential value on the basis of a “better than nothing” or potential educative tool, while at the same time recognizing the limitations of the technology; an encouraging finding considering the commercial interests at play and the potential equity and access issues. Experience with concussion management and concussion-specific policy helped ease feelings of uncertainty among study participants, however, communication breakdowns remained a primary concern of parents resulting in greater uncertainty.

Our findings highlight the need for greater attention to be paid to the specific role and concerns of parents in the concussion management process. Extending from Kroshus and coworkers' (87) study of experts, understanding parent perspectives on what should be reasonably expected in terms of knowledge and skills would be of value in supporting programming for parents. Considering the uncertainty related to parent-child communication, developing a better understanding of the dynamics of parent-child interactions when it comes to communication and integrating a developmental perspective would be of value in guiding parent response. Extending our findings related to parent-coach and parent-physician interactions, understanding the dynamics surrounding injury management within the sporting triad and extending this to urgent and primary care providers as well as other sport participants in concussion management roles [e.g., match officials; (18)], would be valuable in supporting the organization of management and education efforts. Furthermore, parents' experiences can inform future concussion policy and education developments, highlighting the gap in understanding regarding the use of technologies such as NBT and concussion management pathways, leading parents to seek out unproven methods, potentially compromising their child's recovery and welfare.

Theory use has become more prominent in research on parents' response to concussion. We used PMT to support our methods and interpret our findings. Study participants' responses highlight elements of PMT in identifying components of threat (i.e., perceived severity), but also potential modifiers (i.e., achievement-orientated motivation) to threat as well as the role of self-efficacy for identification and management. Response efficacy is reflected in the uncertainty around the use of technology such as NBT. We also found that PMT, as a health cognitions framework, is perhaps more limited as a lens to examine influences such as the role of uncertainty in emotional coping, interpersonal dynamics (e.g., the sporting triad), and larger contextual influences on parent behaviour such as information exchange within sport systems. It may be relevant and interesting to examine parent response through theories that more explicitly capture experiential avoidance, relationship dynamics, and systems influences. Theory informed research should also explore the experiences of key actors in concussion management throughout the entire process, not just the identification and acute management phase. At this point, it may be helpful to “take stock” and review the state of the art of theory use to see where gaps exist to inform future research and intervention development.

This work may also integrate into the larger literature on parent uncertainty with diagnosis, recovery trajectories, prognosis, care environments, and decision making in childhood illness (95) in revealing shared and distinctive concerns with concussion and other childhood illnesses and injuries. Like other research on child illness and injury, uncertainty is a major feature of parents managing SRCs. The antecedents of uncertainty for parents include the inherent limitations in medical diagnostic capacities that they are not made aware of. Communication patterns with children and within the sporting triad and sport systems also undermine parent confidence. Personal experience with concussion and concussion policy help to reduce uncertainty. Consequent to uncertainty, we identify that parents may fall back on management methods that do not have research support and are “better than nothing”. Interventions directed towards parents may benefit from providing opportunities for parents who are managing their child's concussion to connect with experienced peers (e.g., social media contact), given the value attributed to personal and vicarious experiences in managing concussion. Based on this and previous research [cf., (36); and also see (34, 35)], expectations for urgent care assessment and evidence for alternative forms of management and treatment should be made clear to parents in concussion education materials and in consultation with health care providers. Specific to NBT, our findings suggest health care professionals involved in providing NBT should also make clear the potential shortcomings of its inclusion in management as parents are in a vulnerable position where their decisions are driven by understandable worry and concern for their child. Given the demands of parenting, it is unreasonable to expect parents to have expertise in assessing the value of these alternatives.

Finally, our findings and that of other research (87) suggest the need to more centrally involve parents in consultations and research around concussion management, and particularly interventions directed towards their involvement. Parents occupy perhaps the central role in youth sport concussion management. Therefore, their voice is essential in advancing concussion prevention and management efforts.

## Data Availability

The raw data supporting the conclusions of this article will be made available by the authors, without undue reservation.
