# Supplementary material for: Navigating uncertainty: exploring parents' knowledge of concussion management and neuropsychological baseline testing
Source: Front Sports Act Living. 2024 May 10;6:1360329. doi: 10.3389/fspor.2024.1360329 (PMC11116697; doi:10.3389/fspor.2024.1360329)
Supplement: Supplementary file 1 [file Table1.docx]

Focus Group Protocol

Part I- Introduction

Good morning and thank you for participating in our focus group today. We are looking forward to discussing your perceptions and hearing your stories about sport-related concussion and neuropsychological baseline testing. We are also interested in discussing the passages that were emailed to you along with the zoom link.

We will be asking you questions about your knowledge in regard to concussion injury, your past experience dealing with concussion injury in regard to your children and also questions pertaining to concussion management. We would like to remind you that this focus group will be recorded so we would like to ask you to speak clearly and to respect other participants by not trying to talk over them. Please refrain from using gestures and nodding with your head and instead verbalize your responses so they can be detected on our tape recorder. Your responses will also be recorded by Dr. O’Hagan who will be taking the role of the Recorder, is everyone OK with that?

At this time, we would like to remind you that all personal information given in your responses will remain anonymous during data analysis and reporting. We would like to ask you at this time to maintain confidentiality of other participants by not sharing information outside of the focus group. Is everyone OK with that?

We would like to remind you at this time that your participation in this focus group is completely voluntary and we appreciate you giving us your time. At any point during the focus group if you feel uncomfortable or would not like to continue, you are free to withdraw and will receive your compensation.

Part II- Overview

There is currently a lack of information in the research community that looks at how you, the parent, perceive sport-related concussion and the way in which it is managed. This is curious considering that you are the ones who are most involved in your child’s recovery from this injury. Knowing that there is limited understanding on parents’ perceptions of this process, this focus group looks to gather the views from those who have gone through the process of having a child experience a sport-related concussion or have known someone that has. The questions that will be asked during this focus group look to target three main areas of interest. We would like to gain an understanding on how you perceive a sport-related concussion, your experience with sport-related concussion and your views on how they are managed. We appreciate your participation in this group and remember, please speak clearly and try not to talk over others. Thanks.

Part III- Guide

Opening Question

1. Let us open up our discussion by having everyone talk about why they were interested in participating in this study?
2. Could I invite someone to get us started?

Concussion Knowledge

1. What do you know about sport related concussion?
   1. What past education have you received in regard to sport-related concussion? Where did you receive this education?
2. (Perceived severity) In your mind, what are the consequences of sport-related concussion?
   1. Short-term
   2. Long-term
   3. Do you think the consequences are more severe within children?
3. (Perceived occurrence) Is concussion a likely injury for children and youth in sports?
   1. (Perceived occurrence) Do you think that the more involved a child is in sport, the risk of sustaining a concussion becomes greater?
4. (Response efficacy) What measures are effective in reducing the chance of an athlete sustaining a concussion?
   1. Rules?
   2. Equipment?
   3. Skills?

Concussion Experience and Management

1. Do you have any personal experiences dealing with a sport-related concussion regarding your child?
2. How confident do you feel about identifying concussion? What actions did you take to help identify the concussion?
3. What types of management methods did you employ to help with your child’s recovery from sport-related concussion?
4. What do you think might be helpful to provide a parent of young athletes to help them be able to manage a concussion?
   1. What do you feel could have benefitted your child more looking back on the recovery stage?
   2. Is there anything you wish you knew prior to helping your child recover from a sport-related concussion?
5. What did you learn that you did not know prior to your child sustaining a sport-related concussion?
6. How was your experience dealing with medical professionals during your child’s recovery?
7. What measures do you view to be effective in managing your child’s concussion?

Baseline Testing and Media Passages

A type of management process that has been gaining popularity is neuropsychological baseline testing. We sent you media passages with arguments for and against neuropsychological baseline testing. At this time, we would like to review the media passages that we sent you along with the zoom link. We will then focus questions on neuropsychological baseline testing.

(10 minutes to review if need be)

1. Have you ever used the service of baseline testing to help manage your child’s sport related concussion?
   - 1. Why or why not? Was it mandatory? If not, why did you pursue it?
     2. Who provided it?
     3. Who paid for it?
     4. When was it administered?
     5. How was it administered? Rink? Clinic? Home?
2. Review of media passages.
   1. Were the passages easy to comprehend? Do you have any questions about the passages?
3. What are your thoughts on neuropsychological baseline testing?
   1. Do you believe it is a tool that could help manage a sport-related concussion?

We would like to thank you at this time for your participation in our focus group. If anyone has anything they would like to add to our discussion, please feel free to do so now. We are happy to share the results of our research when it is completed.
