# Supplementary material for: Navigating uncertainty: exploring parents' knowledge of concussion management and neuropsychological baseline testing
Source: Front Sports Act Living. 2024 May 10;6:1360329. doi: 10.3389/fspor.2024.1360329 (PMC11116697; doi:10.3389/fspor.2024.1360329)
Supplement: Supplementary file 2 [file Table2.docx]

**Supplementary Material - Media Passages**

The following media passages from the popular press were distributed to participants prior to the focus groups. Technical detail and argumentation were reviewed by experts in neuropsychological testing and philosopy, respectively. Following the initial block of questions concerning general aspects of concussion management, participants were given time to read the passages. Questions concerning neuropsychological baseline testing were then addressed. (See Focus Group Guide for order of questions)

Passage 1 – Argument in support of baseline testing

## New concussion guidelines launched for Canada's Olympians, Paralympians

Typical of a driven athlete, the 28-year-old Olympian from Olds, Alta., tried to shake it off and continue until dizziness and nausea made it impossible. Lappage returned to Calgary to consult with concussion specialist Dr. Brian Benson, who confirmed she was in fact concussed.

A couple of weeks later, Lappage told Benson she was ready to return to the mat. Benson showed her data that said otherwise, in the form of baseline testing conducted when she was fully healthy.

"The baseline test allows you to compare post-concussion to pre-concussion and make sure those numbers are back to their original before you go back to sport," Lappage said. "This baseline test insures you don't go back before you're ready.

"To take that stress off me as an athlete, and to not give me the choice — because I would always choose to go back — to kind of take that out of my hands is actually great for me," she said.

Source: Donna Spencer, Canadian Press

<https://www.ctvnews.ca/sports/new-concussion-guidelines-launched-for-canada-s-olympians-paralympians-1.4341936>

Passage 2 – Argument opposing baseline testing for concussion management

## Is baseline testing for concussions worth it for your kids?

### Some experts doubt procedure's usefulness

"In reality, the vast majority of children and adolescents with concussion can be successfully managed by family medicine physicians and pediatricians without any baseline testing," wrote Dr. Mike Ellis, a neurosurgeon and Medical Director of the Pan Am Concussion Program in Winnipeg, Manitoba.

His clinic does not offer baseline testing. Neither does Concussion North, an Ontario clinic opened by sports medicine physician Dr. Shannon Bauman in 2014 following her own two-year battle with a hockey concussion.

She recommends evaluation by a physician. If treatment is necessary, she says her clinic offers a multi-disciplinary team of experts.

Bauman says she bases her return-to-play decisions on her assessments and those of the experts in her clinic.

She is critical of baseline testing.

Source: Callum Ng, CBC Sports

<https://www.cbc.ca/sports/concussions-baseline-testing-1.3823734>

Passage 3 – Arguments for and against baseline testing for concussion management

## Concussion test draws praise from parents, mixed reviews from doctors

Dr. Kevin Gordon, a pediatric neurologist at the IWK and a professor of pediatrics at Dalhousie University, said baseline testing can be both a good and bad tool.

Gordon said he recommends baseline testing for athletes who play high-risk sports, such as football, and those who are considering turning professional.

“Should it be incorporated into children’s sports? I’m not sure because I’m talking about a high cost with a low probability of something happening. Generally, 85 per cent of concussions recover within one to two weeks,” he said.

Gordon also shares concerns about baseline testing for children, whose results could be non-existent or change dramatically since they are still developing.

But for parents of athletes, like Deidra Saumure, baseline testing is a security blanket.

“He’s young. He has a whole life ahead of him,” she said of her son. “It’s important to me to make sure everything is fine up there.”

As for Brock, he admits he often wonders not if, but when, his next concussion will be.

“All the time. I worry I’m going to fall. I worry I’m going to hit somebody the wrong way. [That] Something’s going to happen,” he said.

The teen admits that besides buckling up his helmet, he isn’t taking many other precautions to ensure he does not get another concussion. But he is confident about getting baseline testing.

“I can see what I’m like before a concussion. I can see what I’m normally like and afterward I can see how far my brain has been damaged or altered to see what kind of issue we’re dealing with.”

“This is my precaution.”

Source: Julia Wong, Global News

<https://globalnews.ca/news/1533002/concussion-test-draws-praise-from-parents-mixed-reviews-from-doctors/>
